# Special Issue “Role of NRF2 in Disease: Novel Molecular Mechanisms and Therapeutic Approaches II”

**DOI:** 10.3390/biom13050813

**Published:** 2023-05-10

**Authors:** Isabel Lastres-Becker

**Affiliations:** 1 Instituto de Investigaciones Biomédicas Alberto Sols (IIBM), UAM-CSIC, 28029 Madrid, Spain; ilbecker@iib.uam.es; 2 Departamento de Bioquímica, Facultad de Medicina, Universidad Autónoma de Madrid (UAM), 28029 Madrid, Spain; 3 Instituto de Investigación del Hospital Universitario de La Paz (IdiPAZ), 28047 Madrid, Spain; 4 Institute Teófilo Hernando for Drug Discovery, Universidad Autónoma de Madrid, 28029 Madrid, Spain; 5 Centro de Investigación Biomédica en Red Sobre Enfermedades Neurodegenerativas (CIBERNED), ISCIII, 28013 Madrid, Spain

This Special Issue (https://www.mdpi.com/journal/biomolecules/special_issues/Nrf2_II (accessed on 9 of May 2023)) focuses on the impact of the transcription factor NRF2 on various systems and its implication in pathologies such as cancer, central nervous system diseases, skin diseases, and periodontitis. Thus, NRF2 is a pleiotropic transcription factor involved in multiple functions. In this Special Issue, we will highlight some of them below.

One of the leading causes of death worldwide is cancer, of which lung cancer has one of the worst prognoses and accounts for approximately 20% of all cancer-related diseases. Kaghazchi et al. [1] showed that NAD(P)H:quinone oxidoreductase (NQO1), a downstream antioxidant gene from nuclear factor erythroid 2-related factors 1 and 2 (NRF1 and NRF2), is upregulated in non-small cell lung cancer (NSCLC) and could be employed as a predictive biomarker. However, they demonstrated that NQO1 expression is high in normal tumor-adjacent tissue, varies depending on the cell type, and is independent of NRF1 and NRF2 in tumors. These data suggest a novel mechanism of action for NQO1 associated with NSCLC. Apart from increased levels of NQO1, it has previously been shown that levels of the transcription factor NRF2 are frequently elevated in many tumor types in patients with poor prognoses. Therefore, new therapeutic strategies are being sought to improve anticancer treatments. In this context, Garufi et al. [2] analyzed the effect of the compound Zn(II)-curcumin on NRF2 activation and its pathway. Zn(II)-curcumin induces the NRF2/p62 signaling pathway, but this activation hinders the cancer cell death sensitivity of this compound. Inhibition of NRF2 potentiates Zn(II)-curcumin-promoted cell death in cancer cell lines. These data indicate the complexity of the pathways involved in cancer processes and the possible therapies to be implemented.

In the last two decades, non-communicable diseases have become the leading causes of death, mainly due to an increase in neurodegenerative disorders. Within these diseases, amyotrophic lateral sclerosis (ALS) and frontotemporal dementia (FTD) are related diseases within the same spectrum, where oxidative stress and neuroinflammation play a fundamental role in the development of neurodegeneration. Therefore, special emphasis has been placed on the possible role of the transcription factor NRF2 in these diseases. Lastres-Becker et al. [3] analyzed the NRF2 signaling pathway both in samples from patients with sporadic ALS and in a transgenic murine model by overexpressing TDP-43^A315T^, both in the motor cortex and the spinal cord. In both cases, the authors observed a significant increase in the NRF2/ARE pathway, indicating that this pathway is upregulated in ALS but is not sufficient to achieve cellular homeostasis. In contrast, in a transgenic model of FTD by overexpression of TDP-43^WT^, no significant changes in the NRF2 pathway are observed, indicating substantial differences in NRF2 between the two pathologies. Differences also exist in other neurodegenerative diseases, such as Alzheimer’s disease (AD), the leading cause of dementia worldwide. De Plano et al. [4] reviewed the role of NRF2 in AD, where there is controversy as to whether this system is increased or decreased in the disease, and its possible use as a therapeutic target. The major clinical trials involving NRF2 in AD and aging are included in this review. Within the context of vascular dementia, spontaneous intracerebral hemorrhage (ICH) following traumatic brain injury can be devastating. Therefore, Loan et al. [5] carried out a comprehensive review analyzing the involvement of the recruitment of microglia and monocyte-derived cells to the area where ICH occurs and investigating the potential role of NRF2 throughout this entire process. Additionally, although to date there are no clinical trials with NRF2 activators in IHC patients, the authors suggested early-phase trials for IHC monitor safety given the older age of this kind of patient.

This Special Issue also includes studies related to skin disorders, such as vitiligo [6] and cornification [7]. Ogawa et al. [6] described the relationship between NRF2 and melanocytes and several pieces of evidence for the association of NRF2 with vitiligo pathology. Interestingly, they highlighted that NRF2 knockdown reduces cultured melanocyte viability, discussed how SNPs in the NRF2 promoter region increase vitiligo risk, and described a list of NRF2 target therapies for vitiligo. Related to cornification, Ishitsuka et al. [7] described an overview of loricrin and its relation to NRF2.

By exploring other aspects of the NRF2 transcription factor, Karaca et al. [8] summarized the recent studies relating probiotics as activators of antioxidant mechanisms through NRF2 in the maintenance of periodontal health. In general, this Special Issue demonstrates the relevance of the transcription factor NRF2 in a broad spectrum of diseases and how its pharmacological modulation can be used as a promising therapeutic target.
